# Post-traumatic growth trajectories among frontline healthcare workers during the COVID-19 pandemic: A three-wave follow-up study in mainland China

**DOI:** 10.3389/fpsyt.2022.945993

**Published:** 2022-08-10

**Authors:** Zhang Yan, Jiang Wenbin, Lv Bohan, Wu Qian, Li Qianqian, Gu Ruting, Gao Silong, Tuo Miao, Li Huanting, Wei Lili

**Affiliations:** ^1^Department of Nursing, The Affiliated Hospital of Qingdao University, Qingdao, China; ^2^Department of Nursing and Hospital Infection Management, The Affiliated Hospital of Qingdao University, Qingdao, China; ^3^School of Nursing, Qingdao University, Qingdao, China; ^4^Department of Neonatology, The Affiliated Hospital of Qingdao University, Qingdao, China; ^5^Intensive Care Unit, The Affiliated Hospital of Qingdao University, Qingdao, China; ^6^Department of Neurology, The Affiliated Hospital of Qingdao University, Qingdao, China; ^7^Office of Director, The Affiliated Hospital of Qingdao University, Qingdao, China

**Keywords:** COVID-19, healthcare workers (HCWs), post-traumatic growth (PTG), resilience, trajectory

## Abstract

**Objectives:**

The COVID-19 pandemic has taken a significant toll on people worldwide for more than 2 years. Previous studies have highlighted the negative effects of COVID-19 on the mental health of healthcare workers (HCWs) more than the positive changes, such as post-traumatic growth (PTG). Furthermore, most previous studies were cross-sectional surveys without follow-ups. This study draws on PTG follow-up during the COVID-19 outbreak at 12-month intervals for 2 years since 2020. The trajectories and baseline predictors were described.

**Methods:**

A convenience sampling method was used to recruit frontline nurses or doctors at the COVID-19-designated hospital who were eligible for this study. A total of 565 HCWs completed the 2 years follow-up and were used for final data analysis. The latent growth mixture models (GMM) was used to identify subgroups of participants with different PTG trajectories. Multinomial logistic regression model was used to find predictors among sociodemographic characteristics and resilience at baseline.

**Results:**

Four trajectory PTG types among HCWs were identified: ‘Persistent, “Steady increase”, “High with drop”, and “Fluctuated rise.” Comparing the “Persistent low” type, the other three categories were all associated with older age, higher education. Furthermore, “Persistent low” was also negatively associated with resilience at baseline.

**Conclusion:**

The PTG of HCWs with different characteristics showed different trends over time. It is necessary to increase the measure frequency to understand the PTG status in different times. Improving HCW’s resilience could help improve staff PTG.

## Introduction

The coronavirus disease 2019 (COVID-19) has taken a significant toll on people worldwide for over 2 years now, and healthcare workers (HCWs) have faced multidimensional challenges in performing their professional duties and responsibilities. Previous studies have focused on HCWs’ psychological trauma and mental distress under the risk of being infected and the heavy workload (1–3). The current literature indicated that HCWs, especially nurses and frontline responders, presented a higher prevalence of psychophysiological stress like anxiety, depression, insomnia, psychological stress, and post-traumatic stress syndrome than the general population (4, 5). The effects of COVID-19 on HCWs’ mental health have been one of the top 10 patient-safety concerns for 2022 according to Emergency Care Research Institute (6). However, aside from the negative changes, the positive effects of COVID-19 on HCWs should also be recognized and assessed (7). The need to develop psychological interventions to help physical and emotional recovery and promote post-traumatic growth (PTG) among HCWs has been highlighted (8).

PTG, developed by Tedeschi et al. (9), indicates that positive psychological changes could be observed as an aftermath of adverse events, which is consistent with the perception of improvements on personal characteristic resources stated in the Conservation of Resources Theory (10). The theory has been used to examine individual responses to environmental stress. When individuals experience stress, they motivate their personal resources, like resilience and emotional expressions, to proactively manage stressful situations (11, 12). Recently, PTG has been examined in various groups in adverse contexts, including frontline HCWs responding to the COVID-19 pandemic. Healthcare providers in public health facilities in Kosovo, with over half of the doctors and nurses had been infected with COVID-19, showed lower level of PTG (*M* = 47.13) than frontline nurses (*M* = 70.53) in designated hospitals in China (13, 14). The level of PTG varies with the characteristics of different groups, like culture, work experience, social support or resilience (12, 15). Previous studies have devoted to the clear understanding of HCWs’ PTG and its influencing factors with cross-sectional design. However, PTG level may changes along with the duration of traumatic event.

Previous studies have suggested that there may be multiple trajectories underlying the course of PTG after a potentially traumatic event such as an earthquake, cancer, or stroke. Regarding PTG following the Wenchuan earthquake in China, four trajectories, including high PTG, increasing PTG, low PTG, and decreasing PTG, were found (16). For adolescents and young adults with cancer, four PTG trajectories, consists of increasing PTG, stable high PTG level, decreasing PTG and stable low level, have been identified during 24-month time spans (17). There are four different kinds of PTG trajectories in young and middle-aged stroke patients, and the initial level and developmental speed of PTG have positive predictive effects on their mental health (18). Several research indicated that PTG is dynamic and heterogeneous between different kinds of groups, which must be considered to better understand the response to prolonged adverse situations (19). And this is fundamental in developing interventions to help nurses’ and physicians’ psychological and emotional health and promoting organizations’ strategic planning and policymaking. Although there are several studies about the current status of PTG among frontline HCWs, none explored the trajectory or changes in PTG during COVID-19 pandemic until now.

This study aims to: (1) describe aggregate trends in PTG of HCWs, (2) identify subgroups with different PTG trajectories, and (3) consider how these trajectories were associated with initial resilience and sociodemographic characteristics.

The period of 2020–2022 was a challenging and unprecedented time for HCWs due to the COVID-19 pandemic, and the stresses brought by the pandemic come on top of risk factors for mental health. Thus, it is worth exploring whether the medical staff and nurses exhibit characteristics of PTG and its trajectory, which could help hospital managers better understand HCWs’ work performance and find effective methods to improve their mental health.

## Materials and methods

### Design and sample

This prospective cohort study was conducted through online surveys on HCWs fighting against COVID-19 from March 2020 to March 2022. A convenience sampling method was used to recruit participants. Inclusion criteria for participants were HCWs (doctors or nurses) in permanent or contracted role, who provided direct or indirect care for patients diagnosed with COVID-19, or employed at the COVID-19-designated hospital. And those were excluded if they were on any type of leave or had pre-existing mental health problems, such as those diagnosed depression, anxiety, or other mental disease. Follow ups on the participants were conducted for 2 years, 12 months apart; this was done through online surveys. Of the 588 staff who responded to the baseline survey and agreed to the follow-up, a total of 565 participants actually completed the 2-year follow-up.

### Participants

The HCWs enrolled are from The Affiliated Hospital of Qingdao University, a prestigious tertiary general hospital with 4,773 beds and consists of five district hospitals located in Shandong province, in the east of China. Since the COVID-19 outbreak in early 2020, the hospital was designated as the provincial and municipal hospital for treating patients with COVID-19. In January 2020, the isolation wards were set up in the West Coast hospital and the first patient with confirmed COVID-19 in Shandong province was admitted. The HCWs were selected and assigned to isolation wards successively due to the increasing number of infected patients. More than 130 doctors and nurses were assigned to Wuhan, the epicenter of this pandemic at that time. In May 2021, the doctors and nurses in this hospital continued to be sent to isolation wards in the Qingdao designated hospital. They were mainly responsible for the isolation care of imported and sporadic cases in Qingdao during that period. In March 2022, Laixi had a COVID-19 outbreak, resulting in the vacation and conversion of the Pingdu district hospital into a special hospital for receiving COVID-19 cases from Laixi (60 km away from Pingdu). Nearly 400 HCWs were assigned in Pingdu isolation wards to take care of patients with COVID-19. The hospital and the HCWs during these three periods were under enormous pressure due to pandemic control and pressures from the government as well as attending to the urgent medical needs of local residents. In addition, the hospital has to undertake strict and heavy pandemic prevention and control measures, such as pre-admission screening of patients, prohibition of visits, closed-loop management of accompanying persons, and massive nucleic acid pharyngeal swab sampling tasks regularly and persistently.

### Measures

#### General information questionnaire

A general information questionnaire designed by our research team was used to collect data on the participants’ sociodemographic characteristics, including age, sex, marital status (single or married), career duration (year), profession (nurse or doctor), religion (whether or not religious), education (college, bachelors, masters), appointment type (permanent or fixed-term), administrative position (managers or not), and the times of frontline support (only once, twice or more). Most of the general information were collected at baseline and the times of frontline support was acquired during the last follow-up on March 2020.

#### Post-traumatic growth inventory

The post-traumatic growth inventory (PTGI), developed by Tedeschi and Calhoun consists of 21 items (20). The revised Chinese version has been used to evaluate PTG in this study. The revised PTGI (the Chinese version) has 20 items in five domains: (1) relating to others (3 items), (2) new possibilities (4 items), (3) personal strength (3 items), (4) self-transformation (4 items), and (5) life perception (6 items) (21). Responses are measured on a scale from 0 (“I did not experience this change as a result of my crisis”) to 5 points (“I experienced this change to a very great degree as a result of my crisis”). The higher the total score, the higher the level of personal PTG. The revised PTGI has good reliability and validity, with Cronbach’s α of 0.874 and the Kaiser-Meyer-Olkin of 0.859 in Chinese trauma patients.

#### Connor-Davidson resilience scale

The Connor-Davidson Resilience Scale was developed by Connor and Davidson (22). The Chinese version of the scale has been used to evaluate the participants’ ability to cope with difficulties or adversities. The scale consists of 25 items examining individuals’ psychological resilience based on the tenacity, strength, and optimism domains. Each item is scored on a scale of 1–5 (1 = never, 2 = rare, 3 = sometimes, 4 = often, and 5 = always). High scores represent high levels of psychological resilience. The Cronbach’s α is 0.890 in the general Chinese population and it is 0.850 in our sample. Since resilience is moldable and may change in time, we only estimate its baseline level and explore its prediction value for PTG among frontline HCWs.

### Data collection

Wenjuanxing^1^ is a popular survey platform in China and was used to develop the present study’s electronic questionnaire. A standardized set of instructions was compiled by the researchers, including information on the purpose and significance of this study. After obtaining permission from the hospital manager and nursing leader, the electronic questionnaires with formatted instructions were sent to the participants who met the inclusion criteria *via* WeChat groups. HCWs were informed that their participation was anonymous and voluntary. Their agreement to participate was expressed by choosing the “I agree” option before filling in the questionnaires, which assures that the respondents have agreed to participate in this survey. The general information questionnaire and PTGI were completed at baseline. PTGI was issued for data collection at 12-month intervals twice in a row.

### Data analysis

We used chi-square (categorical variables) and *t*-tests (continuous variables) to compare the differences between the included participants and those who did not complete the follow-up.

The latent growth mixture models (GMM) was used to identify subgroups of participants with different PTG trajectories. The GMM aids in the identification of subgroups (latent class) of individuals following similar progressions, reflecting differing trajectories of symptoms over time (23). We fitted GMM with an increasing number of latent classes and chose the optimal number of classes based on relative model fit and substantial interpretability. The lower Bayesian information criterion (BIC) was used to determine the better model.

We also considered whether the covariates measured at baseline could predict the trajectory class using multinomial logistic regression model. The latent class was the dependent variable and the sociodemographic characteristics and resilience were the independent variables. The odds of assignment to a particular trajectory class was used to estimate the unit change in each baseline predictor.

IBM SPSS Statistics for Windows, version 22.0 (IBM Corp., Armonk, N.Y., United States) was used to analyze descriptive data and conduct multinomial logistic regression. The GMM model and the forest map of OR value were estimated by R V4.1.2. A two-sided *p*-value of < 0.05 was considered indicative of a statistically significant finding.

### Missing data

**FIGURE 1:**
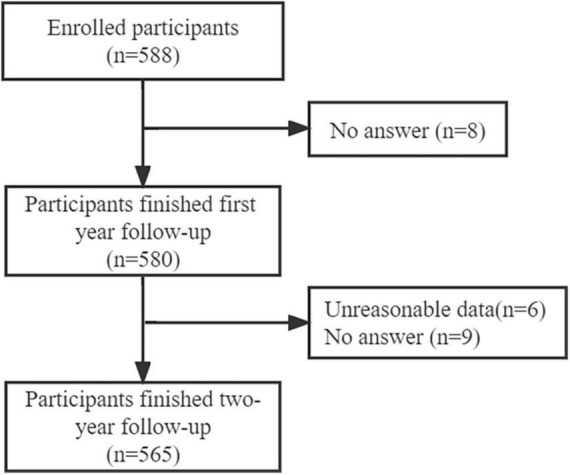
Flow chart of the participants at baseline, 1- and 2-year follow-up.

There were 23 participants of 588 participants (3.91%) who did not complete the 2-year follow-up, 8 of them dropped out during the first follow-up by not answering the questionnaire, and 15 of them were excluded due to either unreasonable information provided about the times of frontline support or no answer at all (Figure 1). The sociodemographic characteristics of those missing date were compared with those included ones to estimate difference.

### Ethical considerations

Ethical approval was obtained. All participants were provided with an explanation of the study aims and procedures before they completed the questionnaires. The completed questionnaires were kept confidential, using file encryption software, and were accessible only to the study authors responsible for data analysis.

## Results

### Cohort characteristics

A total of 565 HCWs completed the 2-year follow-up and were used for the final data analysis. The excluded participants were the younger HCWs (28.74 vs. 31.07 years, *P* = 0.016), those who had a fixed term contract (87.0% vs. 63.9, *P* = 0.023), and those who were in the frontline against COVID-19 only once (100 vs. 69.6%, *P* = 0.002). Among the 565 HCWs, the proportion of female nurses were nearly 90%; less than one-third (30.4%) of the included participants have been in the frontline more than two times. The PTG level was lowest in March 2020 (2.89 ± 1.14), and its tendency was continuously rising in the 2-year follow-up (3.04 ± 0.92, 3.40 ± 0.80). The details are shown in Table 1.

**TABLE 1:** Demographics, resilience, and PTG of participating healthcare workers (*N* = 565).

Demographic		Included	Excluded	*t/*χ^2^*/F*	*P*
		[*n*(%)/*M* ± *SD*]			
Age (years)		31.07 ± 6.58	28.74 ± 4.13	2.580*^a^*	0.016
Sex	Female	497 (88.0)	21 (91.3)	0.024*^b^*	
	Male	68 (12.0)	2 (8.7)		
Education	College	112 (19.8)	8 (34.8)	5.239*^c^*	0.073
	Bachelor	397 (70.3)	11 (47.8)		
	Master or doctor	56 (9.9)	4 (17.4)		
Marital status	Single	254 (45.0)	15 (65.2)	3.656*^c^*	0.056
	Married	311 (55.0)	8 (34.8)		
Religion	No	533 (94.3)	23 (100.0)	0.497*^b^*	0.481
	Yes	32 (5.7)	0		
Nurse/Doctor	Nurse	508 (89.9)	20 (87.0)	0.012*^b^*	0.914
	Doctor	57 (10.1)	3 (13.0)		
Career duration	≤5	242 (42.8)	8 (34.8)	7.102*^c^*	0.131
(years)	6–10	164 (29.0)	11 (47.8)		
	11–15	69 (12.2)	4 (17.4)		
	16–20	59 (10.4)	0		
	>20	31 (5.5)	0		
Job title	Junior	353 (62.5)	13 (56.5)	2.577*^c^*	0.276
	Intermediate	177 (31.3)	10 (43.5)		
	Senior	35 (6.2)	0		
Appointment	Fixed-term contract	361 (63.9)	20 (87.0)	5.153*^c^*	0.023
	Permanent appointment	204 (36.1)	3 (13.0)		
Manager	No	473 (83.7)	23 (100.0)	3.295*^b^*	0.070
	Yes	92 (16.3)	0		
Times of front-line	Only once	393 (69.6)	23 (100.0)	9.897*^c^*	0.002
support	Twice or more	172 (30.4)	0		
Resilience		68.79 ± 16.40	69.61 ± 13.64	−0.281	
PTG*	T1	2.89 ± 1.14		87.57*^d^*	<0.001
	T2	3.04 ± 0.92			
	T3	3.40 ± 0.80			

*PTG level was estimated at baseline (T1, March 2020) and 12-month intervals twice (T2, March 2021; T3, March 2022).

^a^Indicates t-test, ^b^stands for continuous corrected chi-square test, ^c^indicates chi-square test, ^d^for ANOVA.

### Post-traumatic growth trajectories of frontline healthcare workers

**FIGURE 2:**
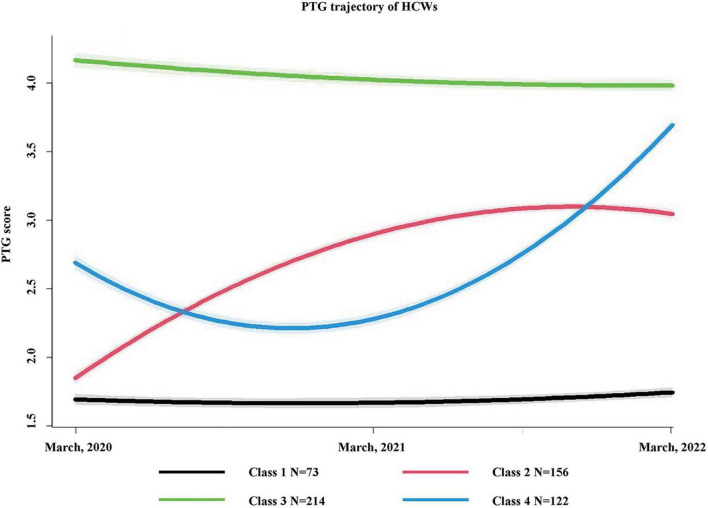
Trajectories of PTG among HCWs from 2020 to 2022. The figure shows trajectories of PTG scores in these 2 years after COVID-19 outbreak among 565 health care workers. Shading around the lines represent confidence bands for the calculated trajectories.

When increasing from four categories to five categories, the BIC value increased from 1509.73 to 1846.86. Therefore, four categories were determined based on the minimum BIC value. Figure 2 presents the four-class PTG trajectories of HCWs.

#### Class 1: “Persistent low”

The “Persistent low” class (*n* = 73, 12.9%) always reported low average PTG scores and remained stable in this low level. The average PTG scores of this class from March 2020 to March 2022 was 1.67 ± 0.15, 1.64 ± 0.14, 1.72 ± 0.14, respectively. The participants showed the lowest level of PTG at the first follow-up; the statistical difference was not significant.

#### Class 2: “Steady increase”

For the “Steady increase” class (*n* = 156, 27.6%), the PTG level showed a trend of steady rise from baseline to the last follow-up (3.06 ± 0.06) (Figure 2). One year after baseline investigation, the participants’ PTG in this class demonstrated a faster increase (Δ = 1.09, *P* < 0.001) and the rate of increase slowed down from March 2021 to March 2022 (Δ = 0.15, *P* < 0.001).

#### Class 3: “High with drop”

The “High with drop” class (*n* = 214, 37.9%) accounted for the most number of participants. The participants showed high level of PTG (4.21 ± 0.42) since the first data collection on March 2020. The PTG also showed a slight downward trend in the following 2 years, yet it still remained at a high level (4.07 ± 0.36, 4.03 ± 0.33, *P* < 0.001).

#### Class 4: “Fluctuated rise”

At the beginning, the participants in the “Fluctuated rise” class (*n* = 122, 21.6%) showed a medium PTG level (2.69 ± 0.06). At the last follow-up, the PTG level was the highest (3.73 ± 0.40), with significant statistical difference (*P* < 0.001). There was a fluctuation in PTG level, which declined to the bottom (2.27 ± 0.18) in March 2021.

### Baseline predictors of different post-traumatic growth trajectories

**FIGURE 3:**
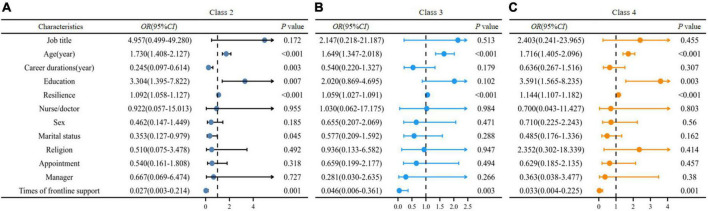
Forest map of multivariate logistic regression. Association of baseline variables with trajectory class assignment (*n* = 565). OR represents odds of class assignment, compared with reference class of “Persistent low.” CI indicates confidence interval. **(A)** Compared with Class 1, the OR value of baseline indicators for Class 2. **(B)** Compared with Class 1, the OR value of baseline indicators for Class 3. **(C)** Compared with Class 1, the OR value of baseline indicators for Class 4.

The multivariate logistic regression was used to evaluate the odds ratio for a unit change in each covariate, and class 1 (Persistent low) was treated as the reference category (Figure 3). For the covariates, age and resilience were continuous variables and the rest variables were all categorical variables. As shown in Table 1, the first of each variable is the reference category.

Comparing the “Persistent low” class with the other three categories, the latter were all associated with older age, short career durations, higher education, and less times of frontline support. When education elevated by one level, the participants were 3.304 times more likely to be in the “Steady increase” group. Simultaneously, the single ones intended to show steady increase of PTG over time. The “Fluctuated rise” class was also associated with higher level education.

For resilience, “Steady increase”, “High with drop”, and “Fluctuated rise” trajectories were all positively associated with high resilience at baseline (*P* < 0.001). That means participants with greater resilience were more likely to exhibit higher levels of PTG over time.

## Discussion

Since the COVID-19 outbreak, HCWs were at risk of infection and experienced high levels of persistent stress, which brings concerns on longer term mental health implications (24, 25). Post-traumatic stress is common during the pandemic and this may affect HCWs’ emotional health. Recently, with the development of positive psychology, researchers have begun to pay attention to the PTG level of medical staff in public health emergencies, but most studies conducted cross-sectional surveys. Over the past 2 years, HCWs as guardians of residents’ health, have repeatedly entered isolation units to care for COVID-19 patients, which may have caused trauma among HCWs (26). This gives rise to questions on how COVID-19 affects HCWs’ PTG, or how the constant stress brought by the pandemic changes their PTG.

To our knowledge, this study is the first to examine the longitudinal changes of HCWs’ PTG during the 2 years since the COVID-19 pandemic. We investigated 565 HCWs (mainly nurses) cohort who were frontliners against COVID-19. As of April 2022, a total of 660 HCWs in The Affiliated Hospital of Qingdao University participated in the frontline support of COVID-19. The medical workers selected in this study could better represent the overall PTG changes.

Using GMM, we identified four classes of PTG trajectories, namely, “Persistent low,” “Steady rise,” “High with drop,” and “Fluctuated rise.” Only 73 (12.9%) of the 565 HCWs were categorized into the “Persistent low” group. The overall PTG trend of both the “Steady increase” and “Fluctuated rise” groups was increasing in the following 2 years despite the fluctuation in the second year (March 2021). Similar to previous studies on PTG and the development process of individuals diagnosed with a disease such as COVID-19 or cancer (19, 27), this result proves that the relationship between traumatic events and PTG is not a single linear relationship. PTG changes over time with significant inter-individual heterogeneity, and this must be considered to better understand the response to prolonged adverse situations and is also crucial for hospital managers to improve the psychological health of HCWs.

Theories on PTG view experienced trauma as the catalyst for fostering lasting personal growth (28). For the participants in the “Persistent low” group, the three COVID-19-related incidents in The Affiliated Hospital of Qingdao University failed to inspire PTG among them. For the “Steady increase” class, each event had played a good catalytic role, and the PTG growth rate is increasing year by year. According to the inoculation theory, individuals who have encountered past trauma may have learned cognitive management strategies making them better able to navigate difficult situations, which is consistent with the trait of their PTG in the “Steady increase” class (29). Although there was a significant decrease in PTG levels in the “Fluctuated rise” class at the first follow-up, there was a more rapid increase in PTG levels at the second follow-up, eventually reaching levels significantly higher than those in the “Steady increase” group. This also indicated that the PTG in this group has strong plasticity and instability. For those in “High with drop” class, they showed a very high level of PTG at the beginning, but with time extension and recurrence of traumatic events, they eventually showed a continuous downward trend, suggesting that hospital managers should also take appropriate measures to maintain the high level of PTG.

Based on this study’s results, the sociodemographic characteristics such as age and education at baseline could predict the PTG trajectories. When age increased by one unit (1 year in this study), the probability of change in PTG level according to the trajectory were 1.730, 1.649, and 1.716 times for the “Steady increase,” “High with drop,” and “Fluctuated rise” groups, respectively, than that of the “Persistent low” group. Previous studies have also shown inconsistent relationships between age and PTG of HCWs in hospitals. Frontline nurses fighting against COVID-19 aged more than 30 years exhibited a higher level of PTG than the younger ones (72.38 ± 16.07 vs. 64.31 ± 17.16, *t* = 3.017, *P* = 0.003) (13). On the other hand, HCWs who experienced the outbreak of the MERS virus in South Korea did not show a difference in PTG levels among different age categories (< 35, 35–49, ≥ 50; *F* = 1.381, *P* = 0.258) (30). The reasons for the discrepancy may be related to population, situation, and age differences. In this study, the higher education of frontline HCWs, the more the changes of PTG tended to follow the other three PTG trajectories (classes 2–4) during the 2 years after the COVID-19 outbreak. This was similar to the results of previous studies on frontline clinical nurses with senior education level who showed a higher level of PTG than those with junior degrees (13, 31).

Except for the sociodemographic factors, resilience was also an important predictor of PTG trajectories. The higher the level of psychological resilience, the lower the probability of PTG showing a continuous low trend (*P* < 0.001). Resilient nurses can cope with stress effectively, persevere in adversities, mobilize responses, and achieve personal growth (3, 32, 33).

Resilience is a skill and trait that may be cultivated by learning resilience-prompting strategies, like cognitive behavioral therapy-based interventions, mindfulness-based interventions or mixed Interventions (34, 35). This finding suggests that hospital managers should cultivate staff resilience, including the ability to respond to public health emergencies to help HCWs achieve higher levels of PTG (36).

### Strengths and limitations

To our knowledge, this is the first study to investigate changes in PTG levels among frontline HCWs over a period of 2 years since the beginning of the COVID-19 outbreak in 2020. Major strengths of the study are the large and fairly representative sample of the provincial and municipal COVID-19-designated hospital, and the high response rate. We also compared the difference between missing data and included data in the analysis. The use of GMM made efficient use of the available data, which was also the *post hoc* grouping method and was better than subgroup analysis.

However, this study also has some limitations. First, convenience sampling was used to select participants. It is likely that the present study sample was not representative of the general HCWs population and the findings may not be generalizable. Second, the distribution of the study participants was imbalanced across nurses and doctors (only physicians). The physicians and nurses work in the same emergency room, and the growth experience may be different (37), which warrants future research. Third, although times of frontline support differed in multivariate categorical logistic regression analysis, we did not analyze its predictive value because of its collection at the last follow-up. Finally, in the 2 years since the COVID-19 outbreak, many experienced medical personnel have been repeatedly assigned to support the frontline. The characteristics of their PTG and predictive value changes over time and must be further explored.

## Conclusion

For HCWs with different age, education levels, and resilience at baseline, the characteristics of PTG trajectories are different. PTG is of great significance for maintaining HCWs’ mental health and promoting positive transformation of negative emotions. We recommend frequently monitoring PTG levels among HCWs and suggest managers to make individualized interventions targeted at populations with different changing trends, which may significantly improve the mental health of healthcare personnel.

## Data availability statement

The original contributions presented in this study are included in the article/supplementary material, further inquiries can be directed to the corresponding author/s.

## Ethics statement

The studies involving human participants were reviewed and approved by Ethics Committee of The Affiliated Hospital of Qingdao University (No: QYFYWZLL27018). Written informed consent for participation was not required for this study in accordance with the national legislation and the institutional requirements.

## Author contributions

ZY carried out the PTG follow-up study, collated the data, and drafted the manuscript. JW, LQ, GR, WQ, GS, and TM participated in the data collection. LB and ZY performed the statistical analysis. WL and LH contributed to the design and supervision of the project. All authors approved the final manuscript.

## Footnotes

1
www.wjx.cn
